# The Prediction of Sinter Drums Strength Using Hybrid Machine Learning Algorithms

**DOI:** 10.1155/2022/4790736

**Published:** 2022-07-07

**Authors:** Xinying Ren, Bing Yang, Ning Luo, Jie Li, Yifan Li, Tao Xue, Aimin Yang

**Affiliations:** ^1^College of Metallurgy and Energy, North China University of Science and Technology, Tangshan, Hebei, China; ^2^Hebei Intelligent Engineering Research Center of Iron Ore Optimization and Ironmaking Raw Materials Preparation Process, North China University of Science and Technology, Tangshan, Hebei, China; ^3^The Key Laboratory of Engineering Computing in Tangshan City, North China University of Science and Technology, Tangshan, Hebei, China; ^4^Yisheng College, North China University of Science and Technology, Tangshan, Hebei, China; ^5^Shanxi Jianlong Industrial Co., Ltd, Yuncheng, Shanxi, China; ^6^College of Science, North China University of Science and Technology, Tangshan, Hebei, China

## Abstract

The prediction model with the sinter drum strength as the evaluation index was established based on the index data and historical sintering data generated during the sintering process. The regression prediction model in the algorithm of machine learning was applied to the prediction of the strength of the sinter drum. After verifying the feasibility of drum strength prediction, different data preprocessing methods were used to preprocess the data. Ten regression prediction algorithms such as linear regression, ridge regression, regression tree, support vector regression, and nearest neighbor regression were used for predicting the sinter drum strength to obtain preliminary prediction results. By comparing the prediction results, the most suitable combinations of data preprocessing algorithms and prediction algorithms for sinter drum strength prediction is obtained. The prediction results show that, for the drum strength of the sinter, using the function data standardization algorithm for data preprocessing has the best effect. Then, using gradient boosting regression, random forest regression, and extra tree regression prediction algorithms resulted in higher prediction accuracy. On this basis, the regression prediction model algorithm parameters are optimized and improved. The parameters of the regression prediction algorithm that are most suitable for the prediction of sinter drum strength are obtained.

## 1. Introduction

A series of complex physical and chemical changes will occur during the blast furnace smelting process. During this process, the composition and quality of the sinter will directly affect the quality, output, and energy consumption of the final smelted product [1]. High-quality sinter is the guarantee for the smooth progress of blast furnace smelting and provides material guarantee for all links in the ironmaking process. The stability of the sinter quality index and the timely adjustment of the process during the sintering process play an important role in reducing smelting costs and promoting energy saving and emission reduction. Sinter is the main raw material for blast furnace ironmaking, and its quality determines the economic benefits of the sinter plant to a large extent and directly affects the production process of blast furnace smelting [2]. The quality indicators of sinter include two aspects: chemical composition and physical and mechanical properties. The chemical composition includes drum strength and reducibility, and the physical and mechanical properties include wear resistance index and soft melting property [3]. The sintering process is a dynamic system with long process flow, many influencing factors and complex mechanism [4]. In the actual sintering work, the traditional sinter drum strength testing is usually to sample and test the finished ore at regular intervals and adjust the sintering process parameters according to the test results. This method cannot perform real-time detection, resulting in untimely feedback of results and inaccurate and untimely adjustment of process parameters in the sintering process. Traditional roasting, parameter setting, proportioning, and other schemes often use linear regulation mode, which often leads to problems such as poor compressive strength [5]. Therefore, the traditional method has more room for improvement, and it is very necessary to predict the quality of sinter in real time.

Through the in-depth study of the formation process of sinter, this paper explores the influencing factors of sinter drum strength and the feasibility of prediction. Then the prediction model of sinter drum strength is established. Different algorithms are used to predict and learn the sinter drum strength. After comparison, the most suitable prediction model for sinter drum strength data is obtained, and finally, the model is optimized. The second section of the article makes an in-depth study on the influencing factors of sinter drum strength. Starting from the interpretation of sinter drum strength, it studies the influence of the liquid generated in the formation of sinter on the drum strength. The material change law affects the formation of liquid phase, as well as the formation of calcium ferrite in the sintering process and its influence on sinter drum strength. It is concluded that the sinter drum strength is predictable. In the third section, the prediction model of sinter drum strength is established, and a variety of data preprocessing algorithms and prediction algorithms used in the model are briefly analyzed. In the fourth section, the data preprocessing algorithm and prediction algorithm, which are most suitable for the sinter drum strength prediction model, are obtained by training the prediction models with different algorithm combinations. The fifth section optimizes the prediction model by adjusting the model parameters on the basis of the best prediction model in the fourth section. The sixth section summarizes and prospects the prediction and algorithm comparison of sinter drum strength.

## 2. Prediction Mechanism of Sinter Drum Strength

In order to realize the prediction of sinter quality, such as drum strength, many scholars have studied the metallurgical properties of sintered ore through experiments and other metallurgical technical means. Liheng Zhang et al. [6] mixed ordinary magnetite and high-chromium vanadium-titanium magnetite (HCVTM) and studied the influence of TiO_2_ content on the properties of HCVTM sinter by sintering cup test. Liang Du [7] found out the quantitative relationship between mineralogicial characteristics and metallurgical properties of sinters by analyzing the influence of import ore on its mineralogicial structure. Xiuli Han et al. [8] quantitatively studied the microstructure of two kinds of high basicity sinters made of magnetite through a polarization microscope and combined with metallurgical performance testing, discussed the influence of the sinter microstructure on its metallurgical properties. Na Yao [9] used mineral phase microscope, XRD, SEM, EDS, and other testing methods to analyze samples and studied the equilibrium phase composition of sinters with different aluminum content and their influence on the metallurgical properties of sinters. Zhengming Yi et al. [10] found in the response surface method optimization study of sinter drum strength that alkalinity and fuel ratio can significantly improve the porosity of sinter, and reducing the porosity of sinter can improve the drum strength of sinter. At the same time, the content of coke and its sintering behavior have a great influence on the final drum strength. Bin Zhang's [11] research found that with the increase of coke content, the chromium content will decrease and the sinter strength will increase, and in order to obtain higher drum strength and yield, the coke content should be kept within a certain range. The quality prediction of sinter requires a variety of computer-based model algorithms, such as regression prediction models in machine learning. Research on these algorithms is the basis for sinter quality prediction.

In recent years, some scholars have also applied intelligent algorithms to basic research on the influence of sintering ore-forming behavior. K Kinnunen et al. [12] used neural networks to analyze data from sintering plant and studied important sintering quality indicators such as optimization of productivity and reduction degradation index (RDI). Wang Ai-min [13] combines the gray theory to weaken the volatility of data series and the advantages of neural network processing nonlinear adaptive information. Using the gray neural network model, the alkalinity of sinter can be accurately predicted using only a small sample. W Chen et al. [14] established a prediction system for sintering chemical composition FeO and sintering yield based on back-propagation (BP) neural network and obtained a high accuracy rate. Through data visualization, Yang et al. [15] studied the relationship between the various components and the compressive strength of the pellet microstructure and provided new research ideas for improving the compressive strength and metallurgical properties of the pellets. However, it is necessary to analyze the sintering process and the calculation of drum strength in detail, and for drum strength prediction data set to use different data preprocessing algorithms and prediction algorithms for algorithms matching.

**Figure 1:**
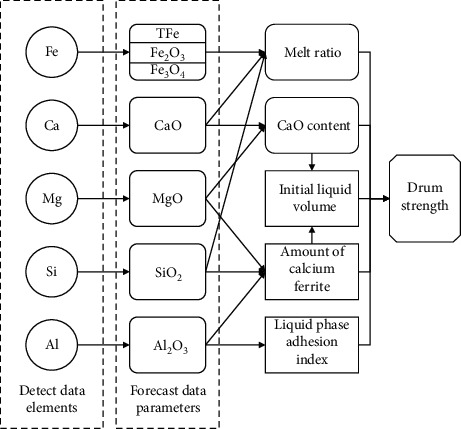
Schematic diagram of drum strength prediction.

To realize the prediction of sinter drum strength, it is necessary to find the relevant factors that have a great impact on sinter drum strength and select the key data for model prediction. Therefore, it is very necessary to study the formation process of sinter and its influencing factors. The ore-forming mechanism of sintering includes three processes: solid-phase reaction, liquid-phase formation, and condensation crystallization. These three processes play an important role in the mineral structure and composition of the final finished ore. Among them, the liquid-phase formation amount of sinter, the liquid phase adhesion index, and the formation amount of needle columnar calcium ferrite are directly related to the final product quality of sinter. The experimental results of Jianfang Wang [16] show that a small amount of Al_2_O_3_ in sinter is conducive to the formation of acicular calcium ferrite and when the ratio of Al_2_O_3_/SiO_2_ is in the appropriate range (0.35–0.40), the quality of sinter will be effectively improved. The four-factor three-level orthogonal experiment designed by Zimin Liu [17] gave the order of the factors affecting the bonding strength: *R*2 > w (SiO_2_) > w (Al_2_O_3_) > w (MgO). The research results of Jian Kang [18] show that the drum strength of sinter decreases with the increase of the fluidity index of the liquid phase and has a greater impact on the drum strength, while under the same experimental conditions, yield and sintering speed are positively related to drum strength. The cohesiveness index of the liquid phase and the behavior during sintering will directly affect the final sinter quality, and in the early stage of sintering, the formation of the liquid phase is affected by other factors: Nan Yang and Xingmin Guo [19] showed that the increase of MgO in the sintering raw material will lead to a decrease in the amount of liquid phase formation in the initial process of sintering heating, that is, the formation of liquid phase is inhibited, resulting in a decrease in the amount of liquid phase. The relevant results are shown in the experiments of Zhengjie Wang and Min Gan [20]. As a result, calcium ferrite changes from plate shape to phase needle column shape, which will effectively improve the sinter drum strength and other indicators. Therefore, based on the factors affecting the drum strength of sintered ore summarized and analyzed earlier, we start from the elements and find the relevant compounds of the corresponding elements in the sintering process. The influence of calcium acid content, the relationship between the influencing factors of the drum strength was drawn. The schematic diagram is shown in Figure 1.

### 2.1. Definition of Drum Strength

(1) $$\begin{array}{c} T=\frac{m_{1}}{m_{0}}\times 100\%. \end{array}$$

In formula (1), *m*_0_ is the weight of the drum sample, in kg, and *m*_1_ is the weight of +6.3 mm particle size fraction behind the drum, in kg. The error requirement is weight of drum sample *m*_0_ and total screening weight after drum (*m*_1_+*m*_2_+*m*_3_). This error cannot be greater than 1.0%, namely:

(2) $$\begin{array}{c} \frac{m_{0}-\left(m_{1}+m_{2}+m_{3}\right)}{m_{0}}\times 100\%\geq 1.0\%. \end{array}$$

When the calculated difference is greater than 1.0%, the drum sample shall be redone, and the allowable difference of drum index is *T*=|*T*_1_ − *T*_2_| ≤ 1.4%. If the allowable difference of drum index exceeds the allowable error value, parallel samples shall be made. If the *T* of supplementary samples meets the aforementioned provisions, a report shall be issued based on the average value of parallel samples.

### 2.2. Liquid Phase Action Mechanism and Adhesion Index

The product obtained by the gradual cooling of the liquid phase in the sintering process is the mineral composition of the sinter and the basis of the consolidation of the sinter. Therefore, the chemical composition, properties, and quantity of the generated liquid phase directly affect the final reducibility and strength of the sinter. The strength of sinter mainly depends on the mineral composition and microstructure. When the liquid phase begins to solidify, the microstructure of sinter begins to form. Owing to the wide temperature range formed by the crystallization process of liquid phase and the recrystallization of solid state, the final mineral composition of sinter is formed in this temperature range [21]. The liquid phase will gradually flow and fill the solid gap of sinter. When the contact area between the liquid phase and the ore core is small, its binding force will be reduced, and there is a large gap between the ore cores, resulting in the reduction of sinter strength [22].

The role of liquid phase in sintering process can be summarized as follows:

1. The unmelted solid ore particles in the sintering process are bonded to each other into blocks and wet their surface. After cooling, the ore particles will be tensioned under the action of surface tension, reducing the gap between solid particles. Therefore, the sinter will have a certain strength after cooling.
2. Owing to the fluidity of liquid phase, viscous and plastic flow heat transfer will be carried out to make the temperature and composition of high-temperature melting zone uniform. After liquid phase reaction, the chemical composition of sinter will be more uniform.
3. In the sintering process, the liquid phase will precipitate new minerals that are not in the sintering raw materials, which is conducive to improving the strength and reducibility of sinter.

The fluidity of liquid phase can be expressed by viscosity. Its viscosity is the internal friction force when the unit velocity difference occurs between two adjacent liquid layers at a unit distance per unit area *η*. The unit is Pa∙s, the viscosity depends on the activation energy of moving particles, and the liquid phase adhesion decreases with the increase of temperature [23]. For homogeneous slag, the effect of temperature on viscosity is as follows:

(3) $$\begin{array}{c} \eta =B_{0}e^{E_{\eta }/RT}. \end{array}$$

In formula (3), *B*_0_ and *R* are constants; *T* is the temperature, in K; *E*_*η*_ is called viscous flow activation energy, which is the activation energy required for a particle to move from one equilibrium position to another. It is related to the structure of composite anion groups in slag.

In addition, the strength of sinter is also related to the internal stress of crystalline minerals formed during sintering and cooling. There are three factors for the generation of internal stress in the condensation process of liquid phase in sintering production [24]:

1. Thermal stress due to the temperature difference between the surface and center of sintered lump ore.
2. The stress between minerals caused by different thermal expansion or contraction coefficients of minerals.
3. The volume expansion caused by polycrystalline transformation of the same mineral produces corresponding stress.

### 2.3. Inhibition Mechanism of MgO on Initial Liquid Phase Formation

MgO is one of the components of sinter feed material, but many studies show that the increase of MgO content will reduce the strength of sinter. First, most CaO react to form CaO–Fe_2_O_3_ during sintering, which is beneficial to the sintering reaction. However, due to the low diffusion rate of MgO, most MgO cannot react with Fe_2_O_3_ and still maintain the mineral state, which is not conducive to the improvement of drum strength [25].

Second, MgO can inhibit the formation of liquid phase. There are two kinds of inhibition: one is that MgO reacts with Fe_2_O_3_ to produce magnesium containing magnetite, and the other is that the addition of MgO leads to the decomposition of CaFe_2_O_4_. Both of them increase the CaO content and promote the formation of high melting point mineral Ca_2_Fe_2_O_5_ and magnesium-containing magnetite. The decrease of CaFe_2_O_4_ content leads to the decrease of liquid phase content in the initial stage, which is not conducive to the sintering reaction [19].

Meanwhile, in the process of solid-state reaction, MgO will promote the decomposition of formed CaFe_2_O_4_ into Ca_2_Fe_2_O_5_ and magnesium-containing magnetite. With the increase of temperature, Fe^3+^ in CaFe_2_O_4_ and Mg^2+^ in MgO diffuse each other at the contact interface between MgO and CaFe_2_O_4_ to form magnesium-containing magnetite. With the continuous diffusion of Fe^3+^ in CaFe_2_O_4_ to magnesium-containing magnetite, the iron content in the adjacent area of the interface between CaFe_2_O_4_ phase and magnesium bearing magnetite phase decreases and the calcium content increases relatively.

### 2.4. Effect of Al_2_O_3_ on Sintering Process and Calcium Ferrite Formation

In the experimental study of Long fang, when the Al_2_O_3_ content is small, the mineral composition of sinter is relatively complex. Increasing the Al_2_O_3_ content can promote the formation of calcium ferrite and inhibit the increase of calcium orthosilicate in sinter, so as to improve the drum index of sinter, continue to increase the Al_2_O_3_ content, increase the glass quality and liquid viscosity in sinter, and inhibit the compactness of the sinter, fine bonding bonds and many pore structures are formed, so that the sinter is subjected to a variety of stresses during cooling, resulting in cracks, resulting in the fragmentation of the sinter, resulting in the deterioration of the sinter drum index [26].

When the Al_2_O_3_ content in sinter increases, the contents of Al, Ca, and Si in calcium ferrite increase significantly. The Al in calcium ferrite increases with the increase of Al_2_O_3_ content in sinter. At the same time, the increase of Al_2_O_3_ content also contributes to the enrichment of Ca and Si in calcium ferrite. However, it should be pointed out that the enrichment state of Al will vary according to the type of ore, which will have varying degrees of impact on the formation and drum strength of needle columnar calcium ferrite. For all minerals containing Al_2_O_3_, the matrix strength of sinter will be reduced with the increase of Al_2_O_3_ content [27].

### 2.5. Effect of SiO2 on Sintering Process and Formation of Calcium Ferrite

In the experimental study under the condition of fixed CaO content and temperature, Shijuan Zhang et al. [28] carried out micro-sintering to study the effect of SiO_2_ content on agglomerate phase. The results show that under the condition of certain CaO content and high binary alkalinity is high enough, SiO_2_ has no significant effect on the formation of calcium ferrite, but plays a decisive role in the morphology of calcium ferrite. When the SiO_2_ content is very low, only massive calcium ferrite can be formed. When the SiO_2_ content reaches 3% to 8%, the needle columnar calcium ferrite interleaving structure can be obtained [23]. Although the formation amount of low-grade sintered cake containing 8% SiO_2_ is very high, its porosity and content are significantly reduced, while the silicate slag phase increases, and the reducibility becomes poor [29]. In general, the increase of sintering SiO_2_ content is beneficial to the occurrence of Si and Ca in calcium ferrite, which can improve the drum strength of sinter.

### 2.6. Conservation Equation in Drum Strength Prediction

When predicting the drum strength of sinter, the invariants in the sintering process can be found according to the sintering mineralization mechanism and the physical and chemical reactions in the sintering process, and then the conservation equation can be established and predicted. Phase change will occur in the sintering process, which is essentially a comprehensive reaction of gas phase and solid phase. The whole phase change reaction should meet the mass conservation equation of both. The mass conservation equation of gas phase and solid phase are shown in (4) and (5) respectively:

(4) $$\begin{array}{c} \frac{\partial \left(\varepsilon \rho_{g}\right)}{\partial_{\tau }}+\nabla \left(\rho_{g}v\right)=\sum_{i=1}^{7}R_{i}, \end{array}$$

(5) $$\begin{array}{c} \frac{\partial \rho_{b}}{\partial_{\tau }}=-\sum_{i=1}^{5}R_{i}. \end{array}$$

In the aforementioned formulas, *i* from 1 to 7 represents the physicochemical changes of water transfer, coke combustion, limestone decomposition, magnetite oxidation, hematite reduction, carbon monoxide oxidation, and the reaction between carbon monoxide and water vapor, respectively. Variables 2 to 5 are gas-solid reactions, resulting in simultaneous changes in the quality of gas and solid. Since the reactions between different gas components will not affect the quality of solid phase, therefore, reactions 6 and 7 only affect the gas phase quality [11]. The gas phase I in the mass conservation equation shall also meet the conservation of matter phase, that is:

(6) $$\begin{array}{c} \frac{\partial \left(\varepsilon \rho_{g}Y_{i}\right)}{\partial_{\tau }}+\nabla \left(\rho_{g}vY_{i}\right)=\sum_{i=1}^{7}R_{i}. \end{array}$$

In addition, due to the complexity of physicochemical changes, phase changes, and energy conversion involved in the sintering process, other conservation conditions should be met. Such as energy conservation conditions in the heat transfer process of porous media, Navier–Stokes equation of incompressible flow field, heat transfer equation between gas and solid phases, and some empirical physical parameter formulas. For the numerical model of drum strength in iron ore sintering process, Bin Zhang et al. [11] have carried out relevant mathematical modeling research.

## 3. Basic Theory of Sinter Blending Model Algorithms

With the development of artificial intelligence technology, more and more researchers apply artificial intelligence algorithms to practical problems. There are many different domains where advanced artificial intelligence algorithms have been applied as solution approaches, such as online learning, scheduling, multobjective optimization, transportation, image processing, and others. Haitong Zhao et al. [30] on the basis of a decomposition-based many-objective optimization framework, a learning automaton (LA) is included in the algorithm, a learning-based algorithm with strong generalization ability is proposed. Junayed Pasha et al. [31] proposed a decomposition-based heuristic algorithm to solve the integrated optimization problem for tactical-level planning in liner shipping, and efficiently tackle large-size problem instances. Zhao Tang et al. [32] studied the machine learning and deep learning algorithms commonly used in the simulation of railway vehicle dynamics and looked forward to the future development direction and key research contents of artificial intelligence algorithms and vehicle system dynamics. Yuannian Qin et al. [33] conducted in-depth research on the theory of ant colony algorithm and its important parameters and studied its application in the fields of job shop scheduling, vehicle routing, image processing, and power system optimization. Maxim A. Dulebenets et al. [34], in order to solve the developed mathematical model and analyze the trade-offs among the conflicting objectives, proposed four multiobjective heuristic algorithms. The developed multiobjective methodology is expected to improve the safety of evacuees at the natural disaster preparedness stage and ensure timely evacuation from areas expecting significant natural disaster impacts. In many different domains, advanced artificial intelligence algorithms have been used as solutions and achieved good application results. The advanced artificial intelligence algorithm also provides a good solution to the engineering prediction problem.

### 3.1. Prediction Algorithms

The so-called prediction is actually to estimate the value of the object requiring solution in a certain state by using historical data. There are many prediction algorithms. The classical machine learning prediction methods include linear regression prediction, nearest neighbor regression, and neural network regression prediction. Classical prediction algorithms show good performance in small sample data prediction. Salminen et al. [35] used classical machine learning algorithms such as logistic regression, naive Bayes, support vector machines, and xgboost to train the model of online hate detection. The accuracy of the model trained by xgboost algorithm is 92%. Arpitmallick et al. [36] predicted the productivity of sintering machine by establishing linear regression and artificial neural network (ANN) models. It is concluded that the prediction of ANN model is better than that of linear regression model. In order to monitor transient Islamic attack in the interior environment and improve risk management of stroke, two machine learning algorithms support vector machine (SVM) and random forest (RF) are used to establish prediction models respectively [37]. The accuracy rate has reached more than 97%. The prediction of sinter drum strength is also a small sample and nonlinear prediction problem. Based on the research on the drum strength of sinter in the second part of this paper, it is feasible to predict the drum strength of sinter by using the chemical composition of the sinter mixture.

In actual sintering production, two types of data, real-time sintering data and historical records, need to be collected to predict the drum strength of sintered ore. Among them, the historical data are mainly used for the training and learning of the prediction model. The real-time sinter data are the data measured by the online detector. The online detection indicators are mainly data related to the chemical composition indicators of the raw materials, such as TFe, FeO, CaO, SiO_2_, Al_2_O_3_, and MgO, the chemical composition of the mixture is used as a prediction model. The input data are input into the trained sinter drum strength prediction model, the drum strength of sinter can be predicted, and then the sinter production can be guided. In this study, the chemical composition of the sintering mixture was used to predict the drum strength of the sintered ore, and the experimental data of the sintering cup was selected to train the prediction model. A total of 243 available data were collected. Examples of experimental data are shown in Table 1.

**Figure 2:**
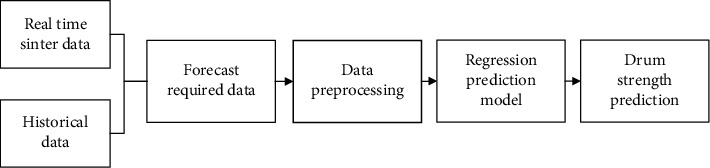
Drum strength prediction process.

The raw data can eliminate the influence of dimensions on model training through data preprocessing. Then use different regression prediction algorithms to train the prediction model to predict the sinter drum strength, and different accuracy can be obtained. The training idea of the sinter drum strength model is shown in Figure 2.

**Figure 3:**
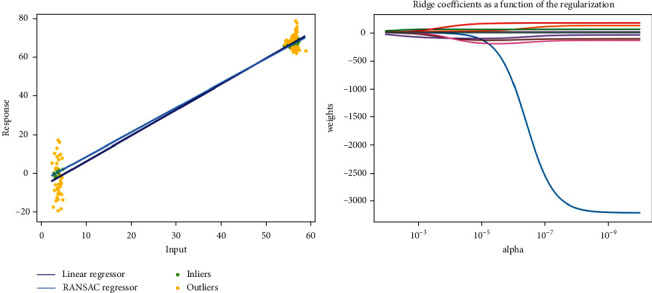
The prediction results of the three algorithms.

Linear regression fits a linear model with coefficients *w*=(*w*_1_,…, *w*_*p*_) to minimize the residual sum of squares between the observed targets in the data set, and the targets predicted by the linear approximation [38]. Ridge regression addresses some of the problems of ordinary least squares by imposing a penalty on the size of the coefficients [39]. The complexity parameter *α* ≥ 0 controls the amount of shrinkage: the larger the value of *α*, the greater the amount of shrinkage and thus the coefficients become more robust to collinearity [40]. When training the model, the training effect of the two algorithms is shown in Figure 3.

**Figure 4:**
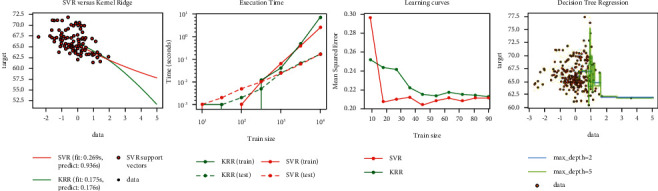
The prediction results of the two algorithms.

The purpose of support vector regression is to obtain a model *f*(*x*) that can fit the training set samples as much as possible [41]. The usual method is to construct a loss function between the sample label and the model predicted value, and minimize the loss function to determine the model *f*(*x*). The regression tree, as the name suggests, is to use a tree model to do regression problems, and each leaf outputs a predicted value [42]. The predicted value is generally the mean value of the output of the training set elements contained in the leaf. When training the model, the training effect of the two algorithms is shown in Figure 4.

**Figure 5:**
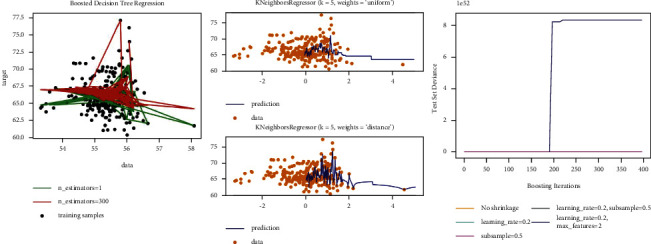
The prediction results of the three algorithms.

In random forests, each tree in the ensemble is built from a sample drawn with replacement from the training set [43]. Furthermore, when splitting each node during the construction of a tree, the best split is found either from all input features or a random subset of size max_features. Random forests achieve a reduced variance by combining diverse trees, sometimes at the cost of a slight increase in bias. The core principle of AdaBoost is to fit a sequence of weak learners on repeatedly modified versions of the data [44]. The predictions from all of them are then combined through a weighted majority vote (or sum) to produce the final prediction. Bagging builds several instances of a black-box estimator on random subsets of the original training set and then aggregates their individual predictions to form a final prediction. Bagging is used as a way to reduce the variance of a base estimator, by introducing randomization into its construction procedure and then making an ensemble out of it [45]. The *K*-nearest neighbor (regression) model is a nonparameter model that uses the target values of the K-nearest training samples to make decisions on the regression values of the samples to be tested. That is, predict the regression value based on the similarity of the sample. Gradient tree boosting or gradient boosted decision trees are a generalization of boosting to arbitrary differentiable loss functions. GradientBoostingRegressor supports a number of different loss functions for regression which can be specified via the argument loss; the default loss function for regression is squared error (‘squared_error'). In ExtraTreesRegressor classes, randomness goes one step further in the way splits are computed [46]. As in random forests, a random subset of candidate features is used, but instead of looking for the most discriminative thresholds, thresholds are drawn at random for each candidate feature and the best of these randomly generated thresholds is picked as the splitting rule [47]. When training the model, the training effect of the three algorithms is shown in Figure 5.

### 3.2. Data Preprocessing Algorithms

Using different data preprocessing algorithms, data can be transformed into data with different characteristics. Using data with different characteristics to train the model, the effect of the model will be different. The data preprocessing algorithm used in sinter drum index prediction model is briefly introduced as follows.

StandardScaler uses the mean and variance to process data that obeys the normal distribution to obtain data that meet the standard normal distribution. MaxAbsScaler transforms a data set composed of vector columns and adjusts each feature to the range of [−1, 1], which is divided by the maximum absolute value in each feature. Min_MaxScaler performs interval scaling based on the maximum and minimum values to convert the data to the 0, 1 interval. RobustScaler uses robust statistics to scale data with outliers (outliers). The QuantileTransformer class scales each feature to the same range or distribution. Performing a rank transformation can smooth the distribution of anomalies and receive less outlier effects than scaling FunctionTransformer constructs a converter based on any callable object. Forward its *X* (and optional *y*) parameters to a user-defined function or function object, and return the result of this function.

## 4. Results and Analysis

### 4.1. Analysis of Prediction Algorithms' Results

For the prediction of sinter drum strength, the variables used in the existing research are not the same. The contents of TFe, FeO, SiO_2_, CaO, MgO, and Al_2_O_3_ of sintering mixture are taken as input variables. The sinter drum index is used as the prediction index. Different prediction models and algorithms are used to predict the sinter drum index. Scaler algorithm is used to preprocess the data, the data are cross-validated according to the ratio of 0.1, and different prediction algorithms are used to predict the drum strength of sinter. The prediction scores of different prediction algorithms are shown in Table 2.

**Figure 6:**
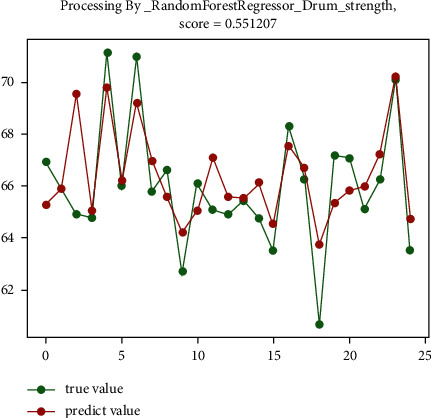
The preliminary prediction effect of random forest regression prediction algorithm.

**Figure 7:**
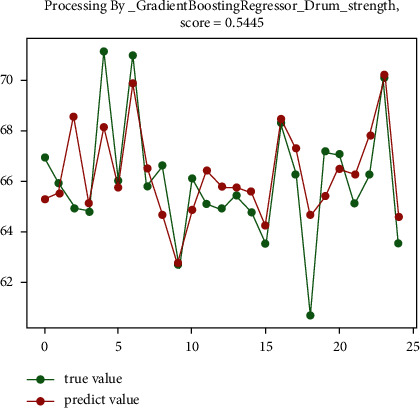
The preliminary prediction effect of gradient boosting regression prediction algorithm.

The analysis shows that for the sinter drum strength, the random forest regression prediction algorithm has the best effect, reaching 55.1%, and the gradient boosting regression prediction algorithm has a relatively good effect, reaching 54.5%. The prediction effect diagram is shown in Figure 6 and 7.

### 4.2. Data Preprocessing Algorithm Result Analysis

When predicting data, different data preprocessing methods will have a greater impact on the results. Therefore, in order to find the most suitable preprocessing algorithm. Different data standardization algorithms are used to preprocess the input variables. The data of sinter drum strength were preprocessed. And further find the most suitable prediction algorithm.

Scaler, Min_max, Max_abs, Robust, Quantile, and Function algorithms are used to standardize the input data of the model. Then different regression prediction algorithms are used to predict the sinter drum strength. The results of the final prediction accuracy are shown in Table 3.

**Figure 8:**
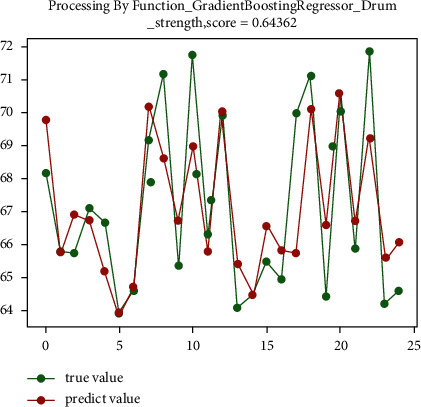
The prediction effect of gradient boosting regression prediction algorithm.

**Figure 9:**
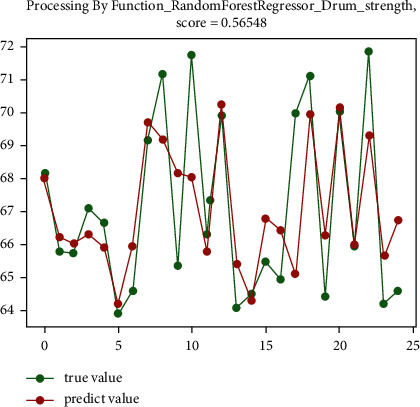
The prediction effect of random forest regression prediction algorithm.

The analysis shows that for the drum strength of sinter, using the function data standardization algorithm for data preprocessing has the best effect. And then using gradient boosting regression, random forest regression, and extra tree regression prediction algorithms, the prediction accuracy rate reaches 64.4%, 56.5%, and 53.2%, respectively. The results of data preprocessing using Robust and Scaler data standardization algorithms are poor, and the results of using regression trees and SVR regression prediction algorithms for prediction are poor. The best prediction effect diagram of drum strength is shown in Figure 8 and 9.

## 5. Optimization Scheme

Various regression prediction algorithms are used to predict the sinter drum strength, and a more suitable algorithm for drum strength data preprocessing and prediction is obtained. The function data standardization algorithm is used to preprocess the sinter drum strength data, and then the gradient boosting regression prediction algorithm is used for regression prediction.

**Figure 10:**
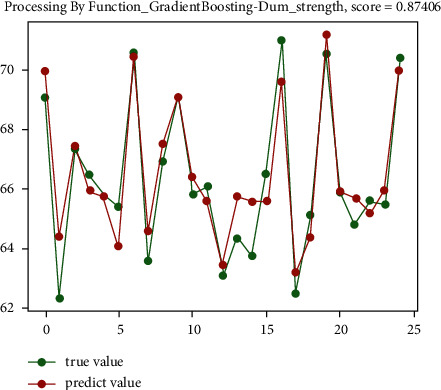
The prediction optimization diagram of drum strength.

According to the prediction results, the parameters of the regression prediction algorithm are continuously optimized and improved to obtain the model parameters suitable for the drum strength. When using gradient boosting regression prediction algorithm to predict the drum strength. The learning_rate is set to 0.03. The n_estimators is set to 350, which can achieve the best prediction effect. The prediction accuracy is 87.4%. The final prediction effect is shown in Figure 10.

## 6. Conclusion and Prospect

Aiming at the prediction of sinter drum strength, this paper deeply probes into the influencing factors and prediction mechanism of sinter drum strength. By comparing 10 regression prediction algorithms and 6 data preprocessing algorithms, the model prediction algorithm and data preprocessing algorithm suitable for sinter drum strength prediction are obtained. The prediction results show that for sinter drum strength, the best prediction effect can be obtained using function data standardization algorithm for data preprocessing and gradient boosting regression prediction algorithm for regression prediction. The experimental results are based on a large number of real experimental data. The compared model algorithms are representative and have practical guiding value for the process control of sintering blending process.

This study uses the composition of the sinter mix to predict the sinter drum strength. At present, the prediction accuracy of the model can reach the demand for guiding sinter production. On this basis, follow-up research can further combine the environment and equipment parameters in the sinter production process to improve and optimize the prediction model and algorithm.

## Figures and Tables

**Table 1:** Example of experimental data for prediction of sinter drum index.

Drum Index	TFe (%)	FeO (%)	SiO_2_ (%)	CaO (%)	MgO (%)	Al_2_O_3_ (%)
71.07	54.71	8.75	5.14	13.05	2.54	2.06
69.87	55.46	9.89	4.89	11.9	2.55	1.79
69.93	55.34	9.66	4.78	12.13	2.5	1.92
70.27	55.23	9.49	4.79	12.31	2.44	1.98
70.6	55.38	9.91	4.78	12.13	2.59	1.95

**Table 2:** Preliminary prediction accuracy of the sinter drum strength.

Algorithm	Accuracy	Algorithm	Accuracy
Linear	0.231	RandomForest	0.551
Ridge	0.257	AdaBoost	0.35
Tree	0.206	G-boosting	0.545
SVR	0.001	Bagging	0.337
Kneighbors	0.392	ExtraTree	0.501

**Table 3:** The prediction accuracy of the sinter drum strength.

	Scaler	Min_max	Max_abs	Robust	Quantile	Function
Linear	0.189	0.158	0.48	−0.081	0.227	0.368
Ridge	0.192	0.137	0.227	−0.075	0.238	0.379
Tree	−1.408	0.178	−0.065	−0.465	-0.68	0.252
SVR	0.112	0.146	−1.586	0.108	0.119	−0.184
Kneighbors	0.182	0.398	0.301	−0.14	0.311	0.38
RandomForest	0.045	0.306	0.239	0.016	0.332	0.565
AdaBoost	0.12	0.141	0.279	−0.077	0.085	0.439
G-boosting	−0.031	0.282	0.404	−0.126	0.158	0.644
Bagging	0.089	0.301	0.316	−0.149	0.039	0.475
ExtraTree	−1.165	0.242	−0.448	−0.407	−0.487	0.532

## Data Availability

The data used to support the findings of this study are available from the corresponding author upon request.
